# The Students of Niagara University Speak

**Published:** 1892-03

**Authors:** 


### THE STUDENTS OF NIAGARA UNIVERSITY SPEAK.

The following action of the class of 1893 of Niagara Universty, on the Exemption Bill now pending in the Legislature, is commended to the consideration of the faculties of the several medical colleges of the State as a model. Like father, like son; like teacher, like pupil. All honor to Niagara University students, say we:

Whereas, The Legislature of the State of New York in 1890 passed an act entitled: "An Act to Establish Boards of Medical Examiners of the State of New York, for the examination and licensing of practitioners of medicine and surgery and to further regulate the practice of medicine and surgery."

And whereas, We are informed that an amendment to Section 11 of said act has been introduced and is now being considered by the present Legislature of the State, the object of which is to exempt graduates of 1893 of all medical colleges in New York State from an examination by or before such Board of Examiners.

And whereas, An amendment to said Section 11, exempting the class of 1892, passed the last session of the Legislature, and the present amendment embodying the same object for the class of 1893, thereby perpetuating same, rendering the law inoperative and of no value.

And whereas, The proposed amendment is applicable to us as the class of 1893, relieving us from the provisions of the above act, but which in our opinion is most unwise; and any act or law which will elevate the standard of medical training, should be hailed with delight by the people of the Empire State, or any State, and also by the professors who fit the students with the knowledge to combat the evils to which flesh is heir.

Now, therefore, We, the class of 1893 of the Medical Department of the Niagara University of Buffalo, N. Y., recognizing the fact that persons intrusted with, and upon whose learning, judgment and skill depends human life, cannot be too well equipped or too well fitted for the most important duties which the physician is called upon to discharge.

And further, Recognizing the wisdom of the appointment of such a Board of Examiners, protest against the proposed amendment to Section 11 of Chapter 507 of the Laws of the State of New York, passed in 1890.

*And be it Resolved,* That we request our representatives in the Legislature to oppose the passage of, and if possible defeat such amendment.

The Class of 1893 by

P. H. HOURIGAN, *President.*

FRED M. BOYLE, *Secretary.*

D. L. DALY,
D. F. WHITE,
H. J. NEWTON,
J. A. BLAIR,
} *Committee on Resolutions.*

